# The role of wind in controlling the connectivity of blue mussels (*Mytilus edulis L*.) populations

**DOI:** 10.1186/s40462-022-00301-0

**Published:** 2022-01-21

**Authors:** Jonathan Demmer, Peter Robins, Shelagh Malham, Matthew Lewis, Aaron Owen, Trevor Jones, Simon Neill

**Affiliations:** 1 School of Ocean Sciences, Bangor University, Askew street, Menai Bridge, LL59 5AB UK; 2 School of Natural Sciences, Bangor University, Bangor, LL57 2DG UK; 3 Extramussel Limited, Refail Llanffinan, Llangefni, Anglesey, LL77 7SN UK

**Keywords:** Lagrangian particle tracking, Larval dispersal, Blue mussels, Connectivity, Ocean model, Irish Sea

## Abstract

**Background:**

Larval connectivity between distinct benthic populations is essential for their persistence. Although connectivity is difficult to measure in situ, it can be predicted via models that simulate biophysical interactions between larval behaviour and ocean currents. The blue mussel (*Mytilus Edulis L.*) is widespread throughout the northern hemisphere and extensively commercialised worldwide. In the Irish Sea, this industry represents ~ 50% of Welsh shellfisheries, where cultivation is mainly based on wild spat. However, the main sources and amount of spat varied interannually (1100 tonnes harvest in 2014 against zero in 2018). The aim of this study is to characterise the structure and dynamics of the blue mussel metapopulation within the northern part of the Irish Sea.

**Methods:**

We develop a Lagrangian particle tracking model, driven by a high-resolution (from 30 to 5000 m) validated unstructured coastal hydrodynamic model of the Irish Sea, to simulate spatial and temporal variability of larval dispersal and connectivity between distinct mussel populations and potential settlement areas.

**Results:**

Our results showed that: (1) larvae positioned near the surface were strongly influenced by wind-driven currents suggesting that connectivity networks had the potential to span hundreds of kilometres; (2) in contrast, larvae positioned deeper in the water column were driven by tidal currents, producing intricate spatial patterns of connectivity between mussel beds over tens of kilometres that were consistent over time.

**Conclusions:**

Dispersal of mussel larvae in the tidally energetic Irish Sea during the April–May spawning season is potentially driven by wind-driven surface currents, as confirmed by fisherman observations of inter-annual variability in wild spat collection. These results have important implications for metapopulation dynamics within the context of climate change and sustainable shellfisheries management (i.e. gain and loss of populations and harvest areas according to wind conditions).

**Supplementary Information:**

The online version contains supplementary material available at 10.1186/s40462-022-00301-0.

## Background

**Fig. 1:**
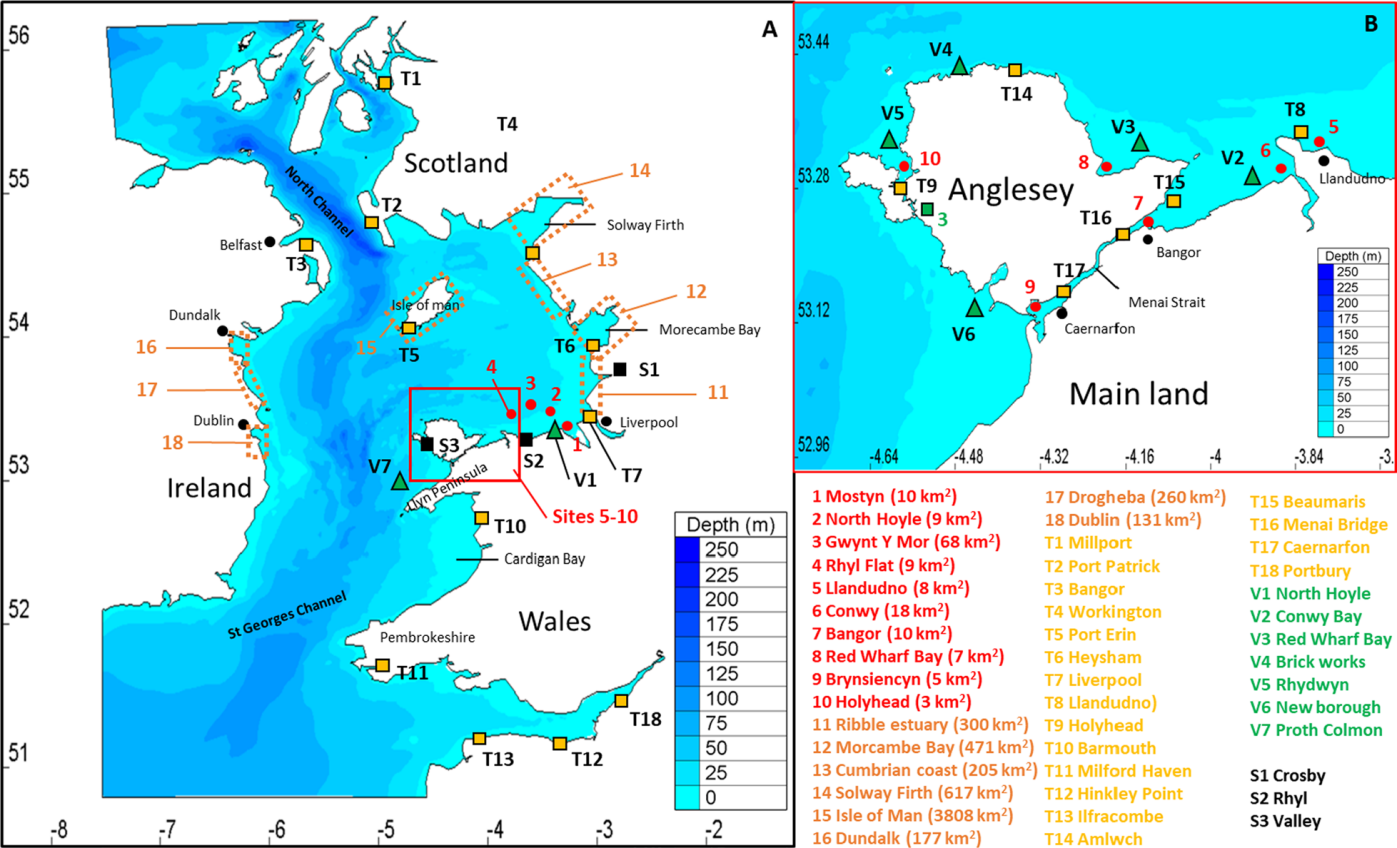
Map of the Irish Sea showing. **A** The hydrodynamic model domain (Lat/lon coordinates) and bathymetry (m rel. to MSL) and **B** the Anglesey area in more detail. The map presents: (1–10) mussel beds denoting larval release and settlement sites (red dots); (11–18) additional coastal larval settlement sites only (dashed orange areas); (S1–S3) meteorological stations used for wind data (black squares); (T1–T18) Tide gauge sites (yellow squares) used for model validation; and (V1–V7) ADCP velocity sites (green triangles) used for model validation. Key oceanographic/geographic regions are shown

Blue mussels (*Mytilus* spp.) have a central ecological importance in intertidal regions [73] by increasing seabed roughness and providing habitat substrate supporting biodiversity of infauna [9, 28, 49]. *Mytilus edulis* are widely distributed across northern Europe, with high densities found off the coasts of Ireland, Wales and France [32]. Mussels are extensively cultivated for food [69], with mussels contributing around 95% of the production and 80% of imputed value to UK shellfish aquaculture, with one third of the industry based in North Wales [34]. *M. edulis* have been successfully cultivated in North Wales for over 50 years (see Fig. 1), the area being suitable due to strong tidal currents through the Menai Strait, that promote the flow of nutrients and water renewal [24, 68]. Mussel production here is based on bottom culture, which uses wild spat collected throughout the Irish Sea that is re-laid on the North Wales beds. Mussel farmers experience interannual variability in wild spat, which directly influences their harvest (pers. com. Trevor Jones), like during the year 2014 (1100 tonnes) and 2018 (zero). It is therefore important to predict the likely dispersal of mussel larvae for efficient stock management and to understand the contribution of the North Wales mussel beds to the wider ecosystem.

Like most benthic organisms, mussels spend their early life stage within the water column, which lasts from 2 to 4 weeks, but it can take up to 10 weeks for larvae to settle at their final location [5, 66]. It has been shown that the pelagic larvae duration (PLD) of mussels in North Wales varies between 20 and 45 days, where sea temperatures range from 8 to 15 °C [7]. Following their pelagic phase, the mussels reach the critical pediveliger developmental stage, when they find a suitable substratum on which to attach [8]. Beyond this basic knowledge, the dispersal of *M. edulis* larvae at present remains largely unresolved and is difficult to measure in situ. Yet larvae defines two fundamental concepts in population biology—*local retention*: the capacity of juveniles to recruit within their parental population; and *connectivity*: the potential dispersal of juveniles between discrete sub-populations [55]. For commercially farmed species, future changes to metapopulations can have both positive (i.e., increased population) and negative (i.e., decline of population) economic impacts. For example, the sustainability of commercial mussel beds over multiple years depends on the local population’s ability to self-recruit larvae each season and on the potential for larval connectivity from surrounding established populations [6, 12].

Potential larval dispersal between discrete populations is controlled by bio-physical interactions between ocean currents and larval behaviour [60]. Ocean currents patterns vary greatly according to tidal dynamics [61], and weather events that modulate density-driven flows and wind-driven currents [71]. Larval dispersal is further modulated by the biological behaviour of larvae: the timing and frequency of spawning events, their growth and metamorphosis rate, PLD, vertical migration behaviour, mortality and settlement behaviour [45]. These dispersal patterns can be consistent over time, e.g. for short duration larval stages [16], but have the potential to vary markedly as a result of a pelagic phase lasting several weeks or due to large variabilities in physical conditions [40, 52].

The circulation in the northern part of the Irish Sea is primarily controlled by an energetic semi-diurnal tidal regime, characterised by large tidal ranges that can exceed 10 m near Liverpool [39]. Tidal velocities are governed by local bathymetry, topography (e.g. headlands) and tidal range, generating regions of strong currents that exceed 2 m/s around islands (e.g. Anglesey) and through the Menai Strait. Weaker tidal currents (< 0.5 m/s) occur in shallower and sheltered bays (e.g. Red Wharf Bay and along the north Wales/English coast) [48]. There is a residual flux northward through the Irish Sea, which is largely tidally-driven [38] and can drive larval transport away from nearshore parental habitats [15, 60]. Within the surface layer, wind-driven currents [41] can further influence larval dispersal, e.g. scallop larvae [35]. Therefore, both barotropic (i.e., tides and wind) and baroclinic (e.g. frontal density-driven flows) currents play a fundamental role in the circulation of the Irish Sea [60] and influence larval dispersal from wild and aquaculture sites off North Wales. However, no studies, to date, have investigated the potential impact of wind-driven currents on mussel larvae in the Irish Sea, including their impact on connectivity.

From the limited published literature on mussel larval vertical distribution, it appears that their position in the water column varies according to several parameters. Dobretsov and Miron [22] showed that mussel larvae are more likely to be at the surface during the last larval stage to increase settlement success. A laboratory study by Sameoto and Metaxas [64] showed that larvae vertically migrate towards food patches or near the surface to avoid high salinity. Knights et al. [42] demonstrated that mussel aggregate in middle and bottom waters during ebb tides but were homogeneously distributed during flood tides—although this could be a consequence of vertical velocities rather than swimming strategy. For this study, we focus on larval dispersal during the early season spawning of *M. edulis*, from March to April, a time period when the water column is well mixed and density-driven currents are weak [33, 39],hence our study can explore the impact of wind field variability on mussel larval dispersal. Density-driven flows develop in the northern Irish Sea where they can have an important role in larval transport [21].

Our aim is to characterise the structure and dynamics of the blue mussel metapopulation within the northern Irish Sea. We hypothesise that the observed natural variability in recruitment of *M. edulis* is influenced by the wind climate. To investigate this, we will apply an Eulerian hydrodynamic model coupled with a Lagrangian particle tracking model to quantify and qualify mussel larval dispersal from selected North Wales mussel populations, covering a range of biophysical scenarios.

## Methods

### Irish Sea hydrodynamic model

The Irish Sea covers approximately 47,000 km^2^ comprising a central channel up to 175 m deep in the west and shallower bays (< 60 m) in the east: Cardigan Bay, Liverpool Bay and Caernarfon Bay (Fig. 1). A hydrodynamic model of the Irish Sea was set up using the Telemac system (V7p2, www.opentelemac.org). The depth-averaged Telemac-2D model is based on the Navier–Stokes equations of momentum and continuity [36]. The depth-averaged configuration of the model is well-suited for this study as the water column remains well mixed for the period of study (March–April) and baroclinic flows are weak [33, 39]. During this early-season period, tidal and wind-driven currents are expected to control larval dispersal. Telemac uses an unstructured mesh configuration and a finite-element solver [75], that can achieve high spatial resolution (e.g. < 50 m at the coast) but lower resolution further offshore. Hence, this configuration is well-suited to resolve strong and variable tidal flows in undulating coastal and intertidal areas, as is the case for our study region. The use of a 3D model would have been computationally challenging to study the Irish Sea at high spatial (< 50 m) and temporal (15 min) scales.

The mesh density, created with Blue Kenue™ grid generation software contains 206,413 computational nodes and covers the entire Irish Sea (Fig. 1), as previous studies show that larvae can potentially travel up to 300 km during their pelagic phase [72]. The spatial resolution of the mesh varied from 30 m in the coastal regions of North Wales, reducing to 5,000 m in deeper offshore regions. The grid was mapped onto bathymetric data, corrected to MSL, comprising an assemblage of: (1) multi-beam data covering shallow regions along the North Wales coast, collected during 2012 (~ 5 m resolution), (2) LiDAR data, covering intertidal regions along the North Wales coast, collected during 2013 (~ 2 m resolution); and (3) Admiralty bathymetric data of the offshore regions (interpolated onto a 200 m horizontal resolution grid) [18]. Thirteen tidal constituents (M2, S2, N2, K2, K1, O1, P1, Q1, Mf, Mm, M4, MS4 and MN4) were applied as forcing at the model’s open boundaries using TPXO7v2, which provides amplitudes of earth-relative sea-surface elevation with a resolution of 1/30° [23]. A constant coefficient of friction of 0.1 was implemented in Nikuradse’s law of bottom friction as it has been used in previous study giving good results on model validation [26].

The hydrodynamic model was used to simulate a two-month period covering March–April 2014 and a three-month period covering March–May 2018, with a computational time-step of 2 s and variables output every 30 min. These periods were chosen based on the period of larval spawning beginning during spring (Jonathan Demmer, personal communication); and observations made from mussel farmers, who noticed in 2014 large mussel settlement in Morecambe Bay (1100 tonnes harvested), whereas in 2018 no settlement was recorded in the same area (Trevor Jones, pers. comm.). Both simulations were initially spun-up for one month (i.e. February 2014 and 2018, respectively). Surface elevations and depth-averaged velocities computed from the 2018 simulation were compared against observations from 14 tide gauges (www.ntslf.org) and seven offshore velocity moorings (data from Bangor University, [47] (Fig. 1). Model accuracy and skill were described using the Root Mean Square Error (RMSE in m for water elevation and m/s for current velocity); the Normalized Root Mean Square Error (NRMSE in %); and linear regression score (R^2^). Tidal analyses were performed on the model data, using the T_TIDE Matlab toolbox, and the principal semi-diurnal lunar tidal constituent (M2 amplitude) compared to observations at 16 sites (taken from Admiralty tidal stream atlas). The validation of the model is presented in Additional file 1: Appendix 1, showing: (1) 5.7% error on simulated tidal elevation on average for 14 sites; (2) 9.8% error on tidal current velocity magnitude; and (3) 11.2% error on tidal current velocity for direction.

### Particle tracking model simulations

A Lagrangian particle tracking model (PTM) was developed to predict the likely dispersal of *M*. *edulis* larvae from ten sites (Fig. 1). These sites represent current commercial mussel beds (Bangor, Brynsiencyn, Holyhead, Mostyn and Conwy), natural mussel beds (Llandudno and Red Wharf Bay) and mussel beds established due to energy infrastructure (Offshore wind farms: Rhyl Flat, Gwynt Y Mor and North Hoyle).

For each simulation described below, 7000 particles were released from each of the ten mussel beds, distributed randomly within an area of 0.2 km^2^. These values were developed through a model sensitivity test [19] to create the most efficient PTM in time whilst simulating enough particles to adequately capture the dispersal variability due to diffusive mixing. Three PTM simulations were performed as presented in Table 1: one simulation representing larvae distributed in the mid-waters (i.e. subjected to tidal currents only) during March–April 2014 (also representing March–April 2018 since the tidal regime was similar); and two simulations representing larvae at/or near the surface during March–April 2014 (Run 2) and 2018 (Run 3). Runs 2 and 3 were repeated with representative wind forcing data from local meteorological stations downloaded from the Centre for Environmental Data Analysis (CEDA): (1) Valley (− 4.52 longitude, 53.25 latitude); (2) Rhyl (− 3.67 longitude, 53.26 latitude); and (3) Crosby (− 2.83 longitude, 53.74 latitude) (Fig. 1). Since the spatial scales of this data were relatively small compared with low pressure systems, linear interpolation between the stations was applied to resolve the spatial variability in wind magnitude and direction at each model time-step.

**Table 1:** Simulations performed to analyse mussel larvae dispersal in the Irish Sea

Simulation ID	Year simulated	Larval behaviour	Meteorological station
Run 1	2014	Mid-water depth	N/A
Run 2	2014	Surface	Valley, Rhyl, Crosby
Run 3	2018	Surface	Valley, Rhyl, Crosby

Surface currents due to wind stress (*U*_*surface_total*_, *V*_*surface_total*_) (Eqs. 1a and 1b) were estimated based on Proctor et al. [57] where: (1) the wind-driven surface current (*U*_*wind_impact*_, *V*_*wind_impact*_, Eqs. 2a and 2b) is assumed to be 3.5% of the 10 m wind speed (*U*_*wind*_, *V*_*wind*_ corresponding to eastwards (x) and northwards (y) components respectively from CEDA); and (2) the barotropic tidal current at the surface (*U*_surface_, *V*_surface_, Eqs. 3a and 3b) is 15% greater than the depth-averaged current speed (*U V*_depth_averaged_, *V*_depth_averaged_) [56]. Ekman transport and Stoke’s drifts have been shown to influence surface currents with the wind stress in the Northern hemisphere [2, 59]. However, Gomez-Gesteira et al. [31] calculated that Ekman transport varied along the coastline. Such data do not exist for the area of interest and so we opted for the simplest approach.

1a $$U_{surface\_total} = U_{wind\_impact} + U_{surface}$$

1b $$V_{surface\_total} = V_{wind\_impact} + V_{surface}$$

2a $$U_{wind\_impact} = U_{wind} \times 0.035$$

2b $$V_{wind\_impact} = V_{wind} \times 0.035$$

3a $$U_{surface} = U_{depth\_averaged} \times 1.15$$

3b $$V_{surface} = V_{depth\_averaged} \times 1.15$$

The timings of particle releases were assumed to occur when the difference between air temperature (AT) and sea surface temperature (SST) was > 2.5 °C in North Wales, which is assumed to induce a spawning event (Demmer et al. in prep). The difference between AT and SST from March 2014 and March 2018 was calculated using data from the Valley meteorological station and a temperature logger continuously submerged that recorded SST near the Bangor mussel bed (− 4.11 longitude, 53.23 latitude) deployed by Cefas (Fig. 1). Results showed a difference of 2.6 °C during the 12 March 2014 and a difference of 3.5 °C the 26th of March 2018 (Demmer et al. in prep). In this study, we assumed that all mussels from each of the ten mussel beds spawned at the same time, representing a mass-spawning event, which is common in spring, rather than trickle spawning events, which are more common later in the season [27].

We simulated larval dispersal using the advective-diffusion scheme represented by Eqs. (4) and (5), which has been applied in previous studies [60, 62]. Equation (4), allows the particle position to be predicted for a future position (${\text{x}}({\text{it}} + 1,{\text{ip}})\;{\text{and}}\;{\text{y}}({\text{it}} + 1,{\text{ip}})$) using initial particle position at time = t, (thus ${\text{x}}({\text{it}},{\text{ip}})\;{\text{and}}\;{\text{y}}({\text{it}},{\text{ip}})$); advection $(({\text{U}}_{{{\text{surface}}\_{\text{total}}}} ) \times \Delta {\text{t}}\;{\text{and}}\;{\text{(V}}_{{{\text{surface}}\_{\text{total}}}} {)} \times \Delta {\text{t)}}$ and diffusivity ($x_{{{\text{diffusivity}}}} \;{\text{and}}\;y_{{{\text{diffusivity}}}}$) for each velocity component (x and y). The random displacement model, for longitudinal and lateral diffusion (*x*_diffusivity_ and *y*_diffusivity_ respectively) in Eq. (5), allows a particle position to be determined based on time step (*∆t*), a random displacement factor (A, between 0 and 1), the standard deviation of Acos(2πA) gives r = 1/√6; and assuming a diffusion coefficient (*K*) of 4 m^2^/s (based on [60].

4a $$x(it + 1,ip) = x(it,ip) + (U_{{{\text{surface\_total}}}} ) \times \Delta t + x_{{{\text{diffusivity}}}}$$

4b $$y(it + 1,ip) = y(it,ip) + (V_{{{\text{surface\_total}}}} ) \times \Delta t + y_{{{\text{diffusivity}}}}$$

5a $$x_{{{\text{diffusivity}}}} = \frac{A}{r} \times \cos (2\pi A) \times \sqrt {(2K\Delta t)}$$

5b $$y_{{{\text{diffusivity}}}} = \frac{A}{r} \times \sin (2\pi A) \times \sqrt {(2K\Delta t)}$$

To represent the continuity of the velocity field, linear temporal interpolation of simulated velocity fields were made from 30 min (Telemac output frequency) to 5 min (PTM timestep) and bi-linear spatial interpolation of velocity data to individual particle positions [65]. Particles that were advected onto land were reflected to their previous position, maintaining the maximum number of particles throughout the simulated period of dispersal [14]. No vertical larval swimming behaviour was simulated as the study focuses on two extreme cases when larvae are transported at the surface (i.e. stronger tidal current and wind-driven current) and at mid-water depth (i.e. weaker tidal current) to define the maximum range of larval dispersal. No mortality was considered as this would reduce the data size for the statistical analyses, and because there is insufficient information on mortality rates during the larval phase.

We simulated five independent PLDs: from 2 weeks (minimal period before settlement; [5] to 6 weeks (maximal period of settlement [20], each with a competency period of one (final) week. The trajectory of each particle was recorded. The simulated trajectories from each wind scenario every year were combined.

### Larval dispersal analysis methods

For each simulated particle, the distance from its source site to its position at the end of each week was calculated using Eq. (6), as a measure of the net transport (NT_i_) cumulated weekly, where t is the time step, n the number of time steps, i is the particle number and *x* and *y* are the particle position. The normality of the data distribution was first studied for all sites. Then an Anova test was performed to study the statistical difference of the distance travelled by larvae depending on the release site and the weekly PLD.

6 $$NTi = \sqrt {\frac{{(x_{i,t1} - x_{i,tn} )^{2} }}{{(y_{i,t1} - y_{i,tn} )^{2} }}}$$

Connectivity and self-recruitment were calculated from week 2 for all simulations, as literature showed that mussel larvae reach settlement stage after 2 weeks in optimal conditions [29]. Connectivity matrices describe the exchange between distinct populations as a percentage of particles reaching a settlement area from a release site [3]. Connectivity has been adapted from the method used in Robins et al. [60] to obtain results in percentage (%). The calculation gave the proportion of larvae that successfully settle (population *i* also called sink population) after the PLD, which originated from population j (i.e. source population) (Eq. 7).

7 $$\left( {{{Tj} \mathord{\left/ {\vphantom {{Tj} {\mathop \sum \limits_{i = 1}^{i = m} pij}}} \right. \kern-\nulldelimiterspace} {\mathop \sum \limits_{i = 1}^{i = m} pij}}} \right) \times 100$$

where *m* is the number of sink populations. Larval wastage is denoted by 1 − *T*_*j*_. In this study, 18 sites were studied: Ten source and sink mussel beds located off the North Wales coast, together with eight settlement only sites further afield in the northern Irish Sea where mussel settlement has previously been observed (pers. comm. with Trevor Jones [Extramussels Ltd] and Nicolas Chopin [BIM in Ireland]) (Fig. 1). Particles were assumed to have settled when they were present within the boundary of one of the 18 sites of interest. Every particle that reached one of the sites of interest during the whole week was counted as a settler. The surface of settlement area was defined according to the site of interest to create deterministic results (Fig. 1).

## Results

### Net transport

**Fig. 2:**
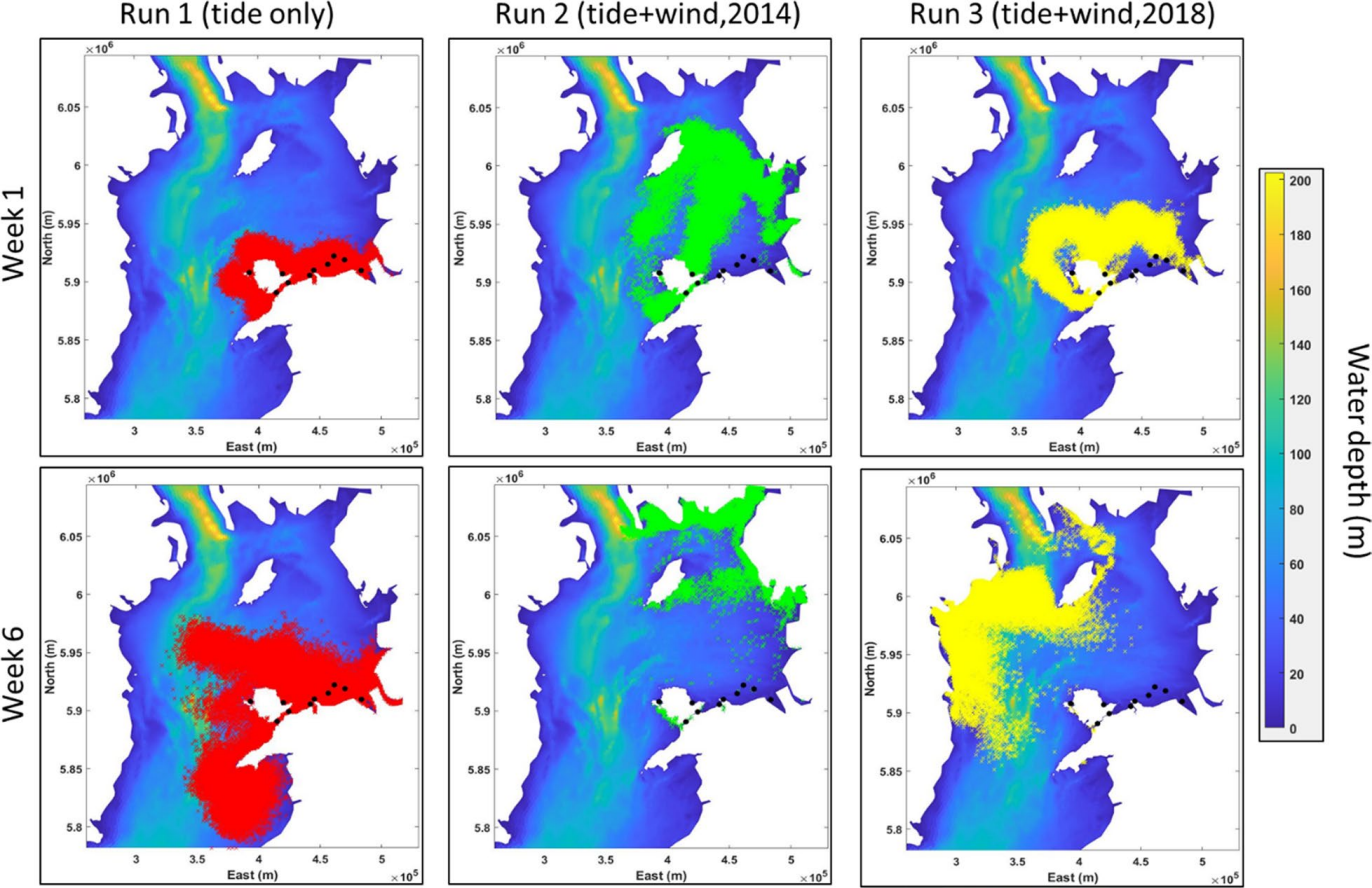
Distribution of particles after 1 week (Week 1: top panels) and six weeks (Week 6: bottom panels) of transport for two larval behaviour: Run 1 where larvae travel at mid-water depth (in red); Run 2 where larvae travel at the surface during March–April 2014 (in green); and Run 3 where larvae travel at the surface during March–April 2018 (in yellow). Results are based from all release sites on 70,000 particles for mid-water depth behaviour (Run 1) and 210,000 particles for surface behaviour (Run 2 for 2014 and Run 3 for 2018). The source sites are represented by black dots

**Fig. 3:**
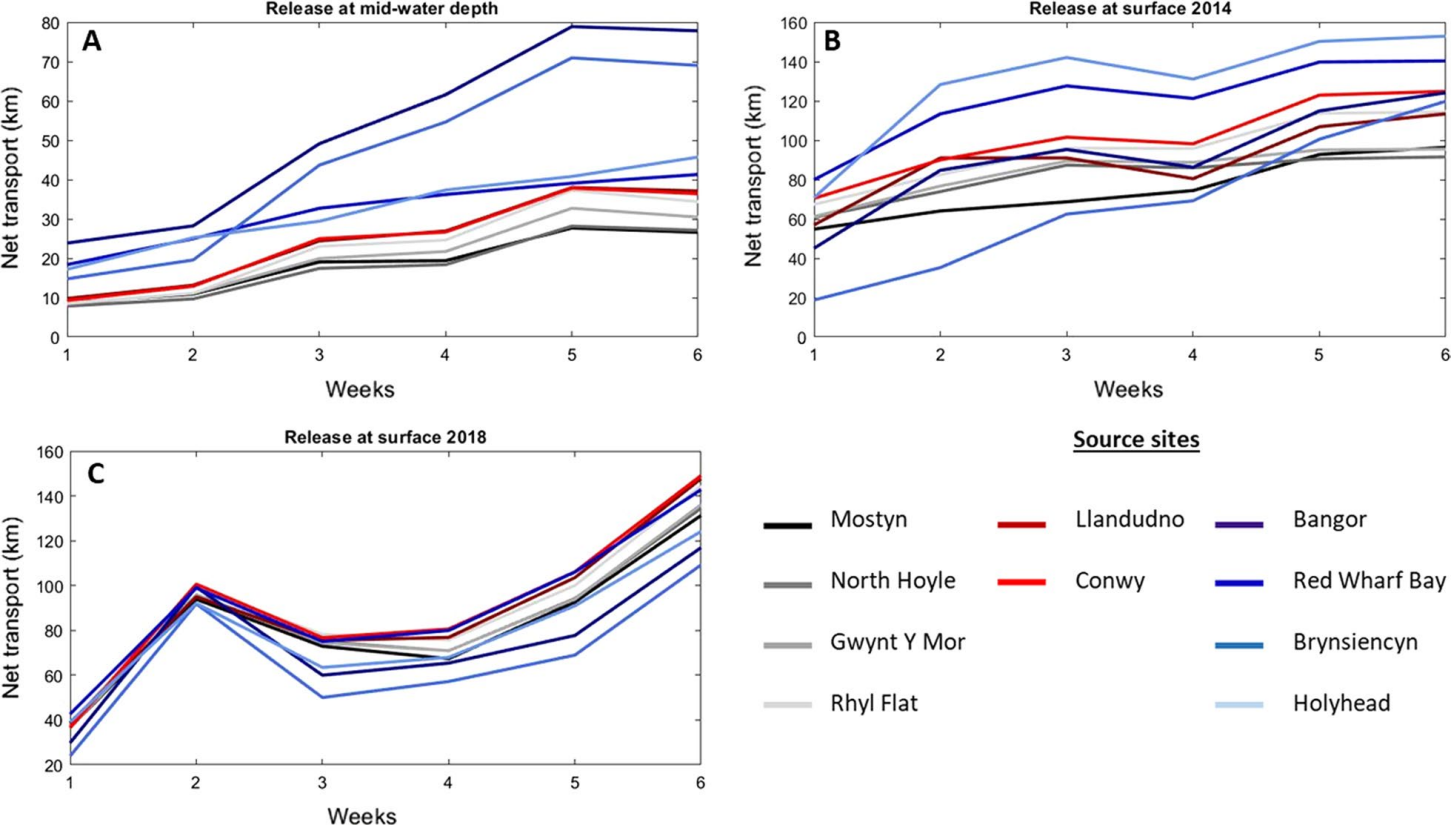
Net transport (km) variation during 6 weeks simulation for particles released at mid-water depth (**A**), at the surface during spring 2014 (**B**) and at the surface during spring 2018 (**C**). Source sites are presented: (1) Mostyn (black); (2) North Hoyle (dark grey); (3) Gwynt Y Mor (grey); (4) Rhyl flat (light grey); (5) Llandudno (dark red); (6) Conwy (red); (7) Bangor (marine blue); (8) red Wharf Bay (dark blue); (9) Brynsiencyn (blue) and (10) Holyhead (light blue)

When simulated larval transport was driven by depth-averaged tidally-driven currents (Run 1), we observed a steady increase over time of the net transport (NT), from 13 km (week 1) to 43 km (week 6), averaged over all sites (Figs. 2, 3). No statistical difference in NT between weeks was observed (Fig. 3A). However, the variability in NT between sources sites increased over time. For example, by week 6, particles from Bangor (North Wales) and Brynsiencyn beds travelled the furthest (73 km on average); particles from Red Wharf bay and Holyhead beds dispersed 44 km, on average and the other source sites (i.e. Conwy, Llandudno, Rhyl Flat, Mostyn, North Hoyle and Gwynt Y Mor) travelled the least (31 km on average) (Fig. 3A).

During the 2014 surface current simulations (Run 2), where particles were dispersed via tide-driven and wind-driven currents, NT increased from 59 km (week 1) to 117 km (week 6), averaged over all particles and sites (Figs. 2, 3B)—i.e. up to a four-fold increase in averaged NT compared with Run 1 (tidal dispersal only). Figure 2 showed that particles are mostly located in the North east part of the Irish Sea at week 6. By week 6, particles from Holyhead and Red Wharf Bay beds travelled the furthest (147 km averaged over both sites); particles from Llandudno, Conwy, Bangor and Brynsiencyn beds and the Rhyl Flat wind farm travelled 119 km on average; and particles from Mostyn beds and wind farms at North Hoyle and Gwynt Y Mor travelled 94 km on average (Fig. 3B).

During the 2018 surface currents simulations (Run 3), where particles were dispersed via tide-driven and wind-driven currents, particles showed an increase in NT from 36 km (week 1) to 134 km (week 6), averaged over all sites (Figs. 2, 3C). Figure 2 showed that particles are mostly located in the western and northern part of the Irish Sea at week 6. The change in NT per week was not steady as seen in Runs 1 and 2; instead, NT increased by 60 km in week 2, averaged over all sites, but then decreased by 26 km in weeks 3 and 4 then increased by 62 km in weeks 5 and 6. The variation of NT through time per source site showed similar trends (Fig. 3C).

The statistical (*T*-test) comparison of NT between treatments (i.e. mid-water depth vs surface) showed a high significant difference (*p* < 0.0001). Furthermore, significant difference was observed between NT at the surface in 2014 and 2018 (*p* = 0.021).

### Self-recruitment

**Fig. 4:**
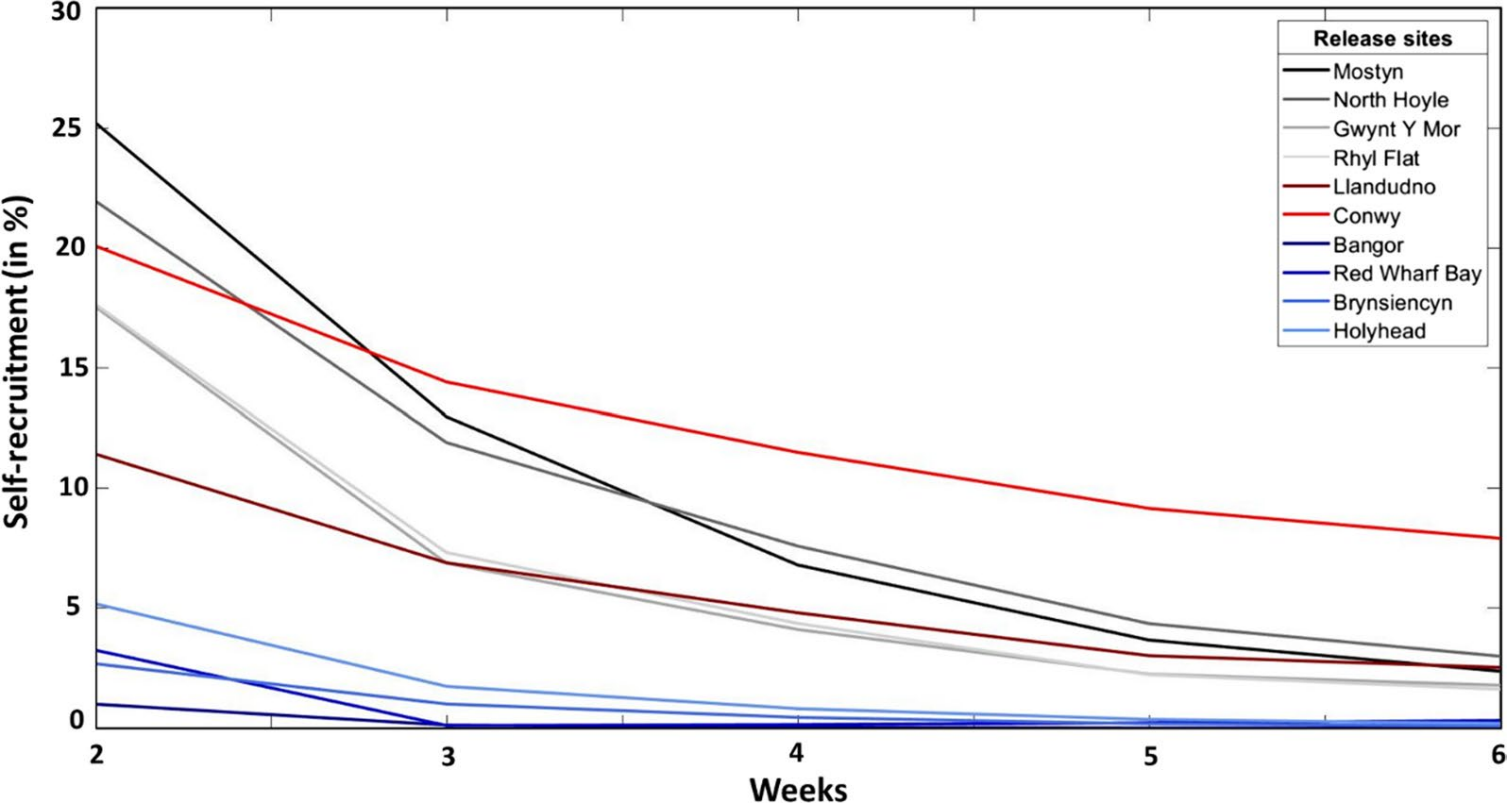
Evolution of self-recruitment (%) during 6 weeks simulation for particles released at mid-water depth (Run 1). Source sites are presented: (1) Mostyn (black); (2) North Hoyle (dark grey); (3) Gwynt Y Mor (grey); (4) Rhyl flat (light grey); (5) Llandudno (dark red); (6) Conwy (red); (7) Bangor (marine blue); (8) red Wharf Bay (dark blue); (9) Brynsiencyn (blue) and (10) Holyhead (light blue)

For Run 1 (tidally-driven dispersal only), simulated self-recruitment after week 1 varied amongst the source sites, being highest at Mostyn (55%) and lowest at Brynsiencyn (10%) (Fig. 4). However, from week 3, minimal self-recruitment was estimated for the Anglesey beds (Bangor, Red Wharf Bay, Brynsiencyn and Holyhead) (Fig. 4). By week 6, Conwy showed the highest self-recruitment (9%) (Fig. 4). The estimated self-recruitment was similar for the Llandudno, Mostyn beds, and the wind farms sites with values of 3 ± 1%, on average. For Runs 2 and 3 (tidal + wind-driven dispersal), self-recruitment was estimated to be generally low (< 1%) for all scenarios, except for the Brynsiencyn bed at week 1 (30% in 2014 and 25% in 2018, data not shown).

### Connectivity

#### Connectivity between mussel beds (sink/sources sites) in North wales

**Fig. 5:**
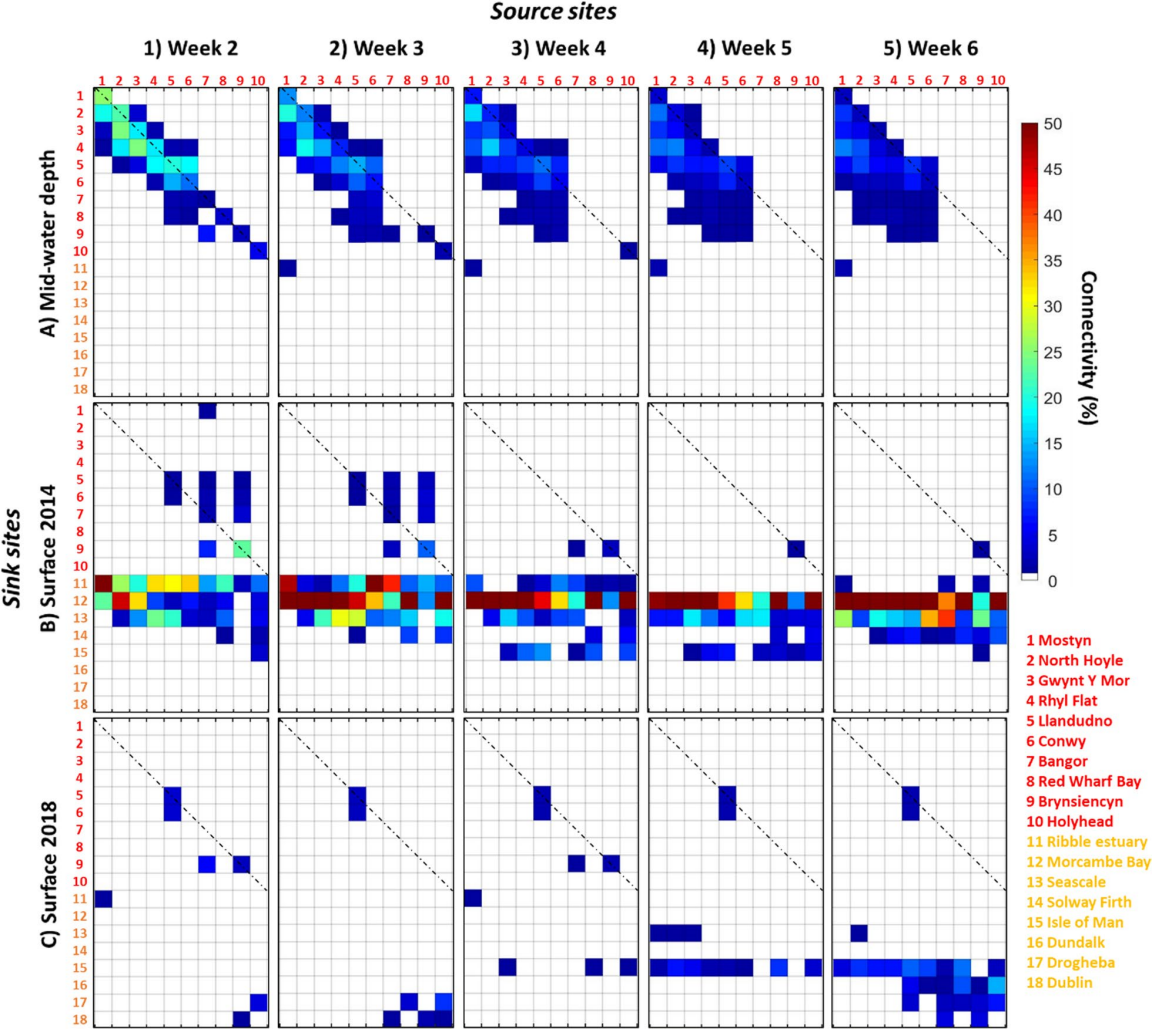
Connectivity matrices for **A** Run 1 (particle released at mid-water depth); **B** Run 2 (particles release at the surface during spring 2014); and **C** Run 3 (particles released at the surface during spring 2018). Connectivity is represented weekly from week 2 to week 6. The colour scale represents connectivity from source (column) to a sink (row); highest values (50%) are coloured in red whereas lowest values (1%) are coloured in dark blue. The absence of connectivity (0%) is represented in white. Retention within the release sites are highlighted by cells that crossed the diagonal dashed line

For Run 1 (tidally-driven dispersal only), the sites located on Anglesey (Holyhead, Red Wharf Bay, Brynsiencyn and Bangor) did not show connectivity from week 4 (Fig. 5A.3). Red Wharf Bay, acted as a sink from week 2 to week 6 with a constant connectivity value of 1.8 ± 0.5% but the number of source sites connected increased from 2 (Conwy and Llandudno) at week 2 to 5 (Conwy, Llandudno and the wind farms) at week 6 (Fig. 5A.5). Furthermore, Holyhead did not show any connectivity with the other sites located in North Wales (Fig. 5A). Conwy and Llandudno showed high connectivity together as both source and sink site, however this connectivity decreased from week 2 to week 6 (Fig. 5A). Mostyn did not act as sink site, however it showed connectivity as a source site with one site at week 1 (North Hoyle: 8.4%) and 4 sites at week 6 (the wind farms: 9.2 ± 3% and Llandudno: 5.9%; Fig. 5A.5).

Next, Run 2 (tidal + wind-driven dispersal in spring 2014) showed no connectivity between Mostyn, Red Wharf Bay and Holyhead as both source and sink site during the period simulated (Fig. 5B). Furthermore, no connectivity was estimated for all sites as both sink and source during week 5 and week 6 (Fig. 5B.4, B.5). Brynsiencyn acted (1) as source with Conwy and Llandudno during week 2 and week (2) as sink with Bangor at week 4 (1%) (Fig. 5B). The wind farms (North Hoyle, Rhyl Falt and Gwynt Y Mor) showed no connectivity from week 2 as both source and sink sites (Fig. 5B).

Finally, Run 3 (tidal + wind-driven dispersal in spring 2018) simulated no connectivity from week 3 between sites located in North Wales as both source and sink (Fig. 5C). Conwy showed connectivity with only Llandudno from week 2 until the end of simulation (2%, on average for all week) (Fig. 5C).

The comparison between the three runs showed that wind-driven currents (Run 2 and Run 3) reduce the connectivity among local populations located in North Wales. Consequently, the larvae have been transported to areas further afield.

#### Connectivity between commercial mussel beds and natural coastal beds

When the particles dispersed at mid-water depths (Run 1), no connectivity was simulated from sites 1–10 with any of the eight additional sink sites: Morecambe Bay, Ribble estuary, Cumbrian coast, Solway Firth, Isle of Man, Dundalk, Drogheba and Dublin (Fig. 5A). For Run-2 and Run-3, connectivity was estimated from some source sites with the additional English and northern sink sites, but not with the Irish coast sites 16–18 (Fig. 5B). Indeed, Morecambe Bay and the Cumbrian coast showed connectivity with all source sites from week 2 (Fig. 5B.1). Results showed a constant increase until week 6 reaching: (1) 50.7% for Morecambe bay; (2) 20.3% for the Cumbrian coast; and (3) 5.2% for the Solway Firth, averaged over all source sites connected (Fig. 5B.5). Estimated connectivity to the Ribble estuary showed highest connectivity during week 2 (24.6%, averaged over all source sites connected), then decreased until week 6 to reach 2% (Fig. 5B.5). Connectivity to the Isle of Man was highest (7.8%) during week 4. However, no connectivity was observed with the Irish coast (Fig. 8B).

A markedly different pattern of connectivity was predicted for Run 3 (the spring 2018 surface current simulation) than during Run 1 and Run 2. During 2018, surface particles were advected towards the Irish coast during week 2, week 3 and week 6 from source sites located on Anglesey (Fig. 5C). However, this connectivity varied through time as it was not simulated during weeks 4 and 5 (Fig. 5C.3, C.4). Simulated connectivity to the Isle of Man reached highest value (6.8%) during week 6, averaged over all sources sites connected (Fig. 5C.5). No connectivity was simulated with the English coast sink sites (Morecambe Bay, Ribble estuary and the Cumbrian coast) (Fig. 5C).

### Contribution of North Wales mussel aquaculture to wider populations

During the spring 2014 surface current scenario (Run 2), our results estimated potential connectivity over 6 weeks from all 10 source sites to the central and eastern settlement sites: Ribble estuary, Morecambe Bay, the Cumbrian coast and the Isle of Man (Fig. 6.1). The highest connectivity was from Mostyn to the Ribble estuary (27%). Morecambe Bay showed relatively high connectivity with neighbouring source sites: Mostyn and the wind farms (13.5% ± 2%, averaged for the four sites). The Cumbrian coast showed highest connectivity (14.5% ± 3, on averaged for the 6 sites) from beds at Bangor, Conwy, and Llandudno, and wind farms at Rhyl Flat and Gwynt Y Mor (Fig. 6.1). The Solway Firth showed connectivity from seven source sites, mainly beds at Red Wharf Bay and Holyhead (34.5% ± 3, on averaged for the two sites) (Fig. 6.1). The Isle of Man connectivity comes from seven source sites, mainly from Llandudno and Holyhead beds (24.5% ± 2, on averaged for the two sites).

**Fig. 6:**
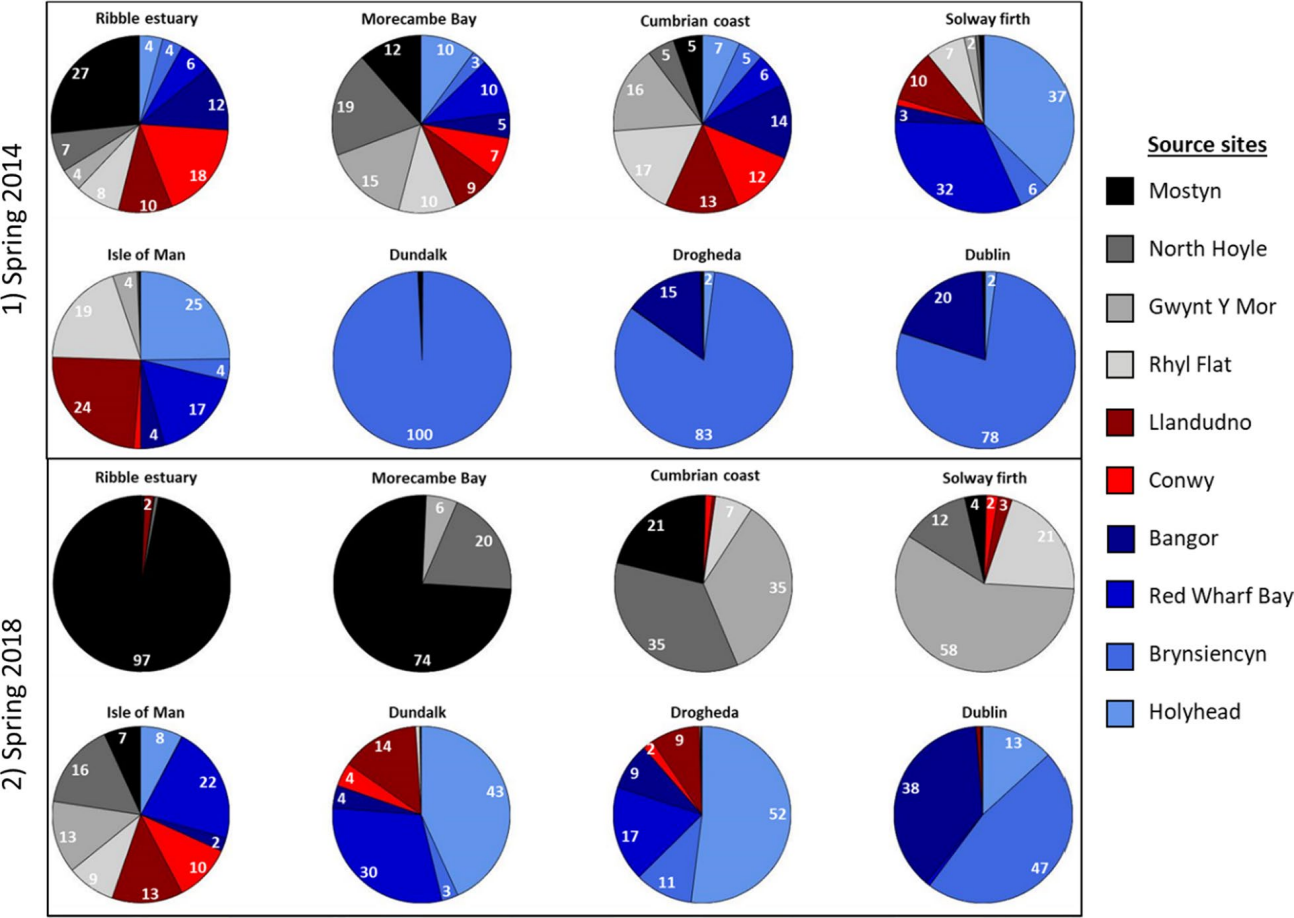
Contribution (%) of source sites located in North Wales to sink sites located elsewhere in the Irish Sea for larvae dispersal simulated at the surface during (1) spring 2014 and (2) spring 2018 on average for all weeks. Sources sites are (1) Mostyn (black); (2) North Hoyle (dark grey); (3) Gwynt Y Mor (grey); (4) Rhyl flat (light grey); (5) Llandudno (dark red); (6) Conwy (red); (7) Bangor (dark blue); (8) Red Wharf Bay (marine blue); (9) Brynsiencyn; and (10) Holyhead. Contribution are labelled in white inside the corresponding area. Contribution of source sites ≤ 1% are shown but not labelled

During the spring 2018 surface current scenario (Run 3), our results again simulated potential connectivity over 6 weeks from all or some source sites to the surrounding coasts along England, Scotland, Isle of Man and Ireland (Fig. 6.2)—but in markedly different proportions to the 2014 simulation. The English and Scottish coasts (Morecambe Bay, Ribble estuary, Cumbrian coast and Solway Firth) showed connectivity primarily from Mostyn bed and the three wind farms (Fig. 6.2). The Ribble estuary and Morecambe Bay showed highest connectivity from Mostyn bed (97% and 74% respectively). Simulated connectivity to the Cumbrian coast was primarily from North Hoyle and Gwynt Y Mor wind farms (both 35%). Gwynt Y Mor was highly connected to the Solway Firth (58%). The Isle of Man showed potential connectivity with all sites excepted the Brynsiencyn bed with highest connectivity from the bed at Red Wharf Bay (22%). The Irish coast was mainly connected from Anglesey beds, the highest connectivity being: Holyhead → Dundalk (43%), Holyhead → Drogheda (52%), and Red Wharf Bay → Dublin (47%) (Fig. 6.2).

## Discussion

Inter-annual variability in larval dispersal and connectivity of wild and farmed mussel populations in the northern Irish Sea was investigated for two contrasting years. The parameterisation of 2D wind-tide flows and larval behaviour were explored by comparing depth-mean transport to surface current transport. Indeed, our experiments represented two plausible larval dispersal scenarios: larvae positioned at/near the surface transported by tides and wind, or larvae positioned in mid-waters and transported by tidal currents only. Our surface-larvae scenarios encountered markedly different wind conditions that occurred during spring 2014 and 2018, when *M. edulis* larvae are thought to predominantly spawn [46, 51].

Model scenarios were designed following observations made by North Wales mussel farmers of interannual variability of seed recruitment in the Irish Sea, which could be the consequence of biological (larval behaviour) and/or physical parameters (e.g., wind-driven flow). For example, previous studies have shown the importance of circulation patterns on interannual variability of larval recruitment and dispersal [44, 54, 74], and larvae located at/near surface have been shown to encounter increased dispersal compared with deeper larvae [44, 50, 76]. Interactions between larval vertical migration and stratification has been shown to be an important driver of dispersal [58]. Although the northern Irish Sea remains well mixed each year until around May [39], which implies that stratification might not play a role in our study on larval dispersal.

In our model study, virtual larvae distributed in mid-waters dispersed away from their natal bed by approximately 33 ± 10 km after 4 weeks and 43 ± 10 km after 6 weeks, suggesting that this proxy behaviour allows for local connectivity rather than with distant beds more than 50 km away. On the other hand, our simulations with larvae transported at the surface estimated a net dispersal significantly higher (82 ± 30 km after 4 weeks and 125 ± 30 km after 6 weeks). These results suggest that the blue mussel populations in the Irish Sea could be highly connected for either behavioural scenario.

Assuming first that mussel larvae are distributed throughout the water column, e.g., developing weak vertical migration and in the absence of stratification, then their dispersal would be primarily controlled by tidal currents and in particular tidal residuals pathways [58]. Tidal residuals do not change markedly from one month to another or between years [37]. Under this scenario, larvae from the Bangor and Brynsiencyn beds dispersed south-westwards through the Menai Strait, towards the Llyn Peninsula and into Cardigan Bay—in accordance with the tidal residuals [10]. No connectivity was estimated with the Irish coast or northern Irish Sea—instead, connecting with mid-Wales populations. Larvae from Red Wharf Bay and Holyhead dispersed to the middle of the Irish Sea following the strong residual currents [61]. The weak residual current in Liverpool Bay explained why larvae from the wind farms (North Hoyle, Gwynt Y Mor and Rhyl flat) remained within Liverpool Bay as previously observed by Davies and Aldridge [17]. However, future studies could examine larvae located in the mid-water column in more detail, as Ekman transport can influence the entire mixed layer and so impact larval dispersal [53].

On the other hand, assuming that mussel larvae are mainly distributed in the surface waters, then their dispersal would additionally be influenced by wind-driven currents. Larvae positioned at the surface dispersed up to 160 km within 6 weeks (Fig. 3B, C). The Irish coast and Morecambe Bay are approximately 100 km from the source sites. The Isle of Man is 75 km away and the Scottish coast is approximately 150 km away. North Wales mussel larvae in surface waters can, therefore, potentially settle anywhere along these coasts and contribute to a metapopulation in the northern Irish sea [11, 63], although we found noticeable differences due to variability in wind direction. During their PLD, *M. edulis* larvae can potentially settle on debris and macroalguae present in the water column as presented by Alfaro et al. [1] for green-lipped mussels. Consequently, the PLD could be greater than 6 weeks and spat could settle in areas further away.

**Fig. 7:**
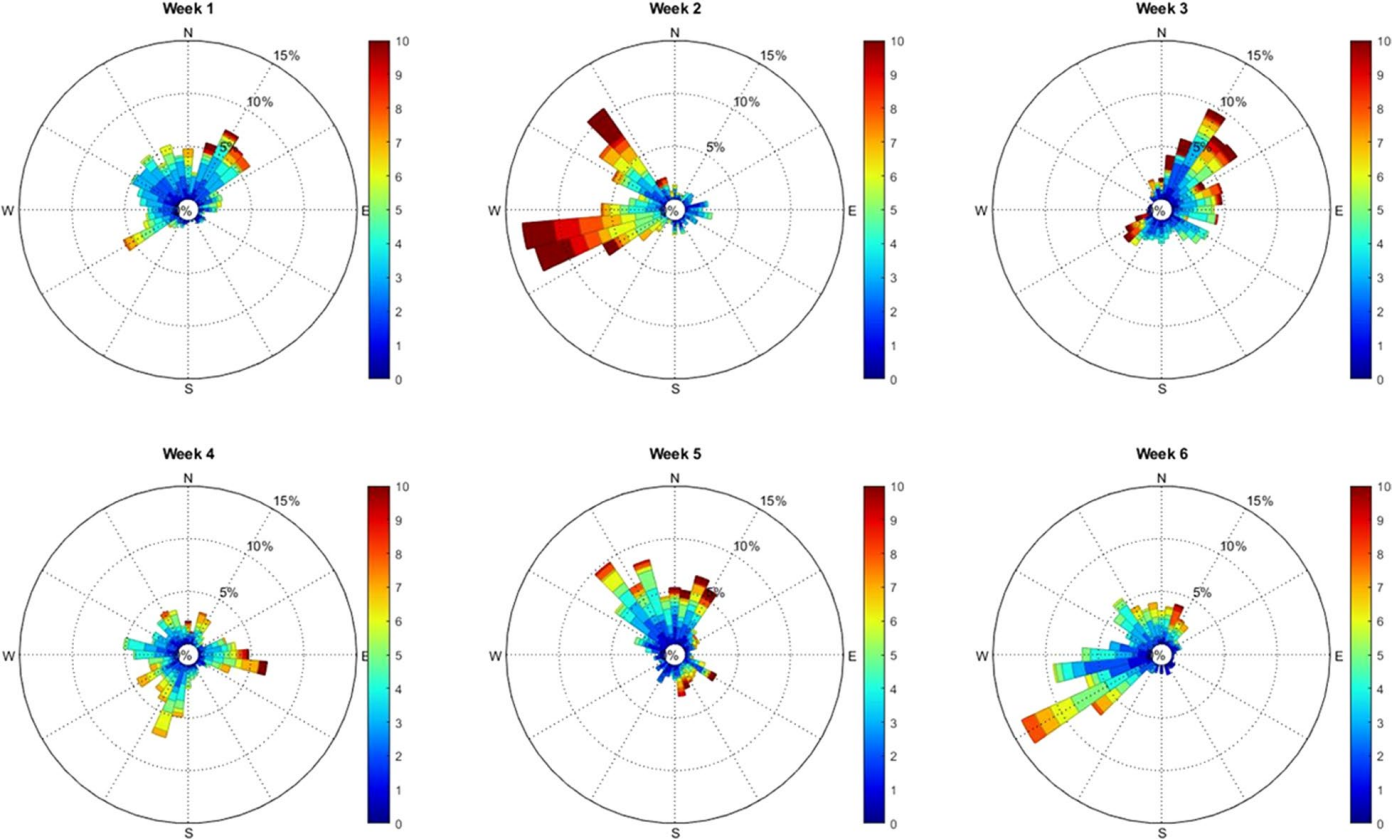
Wind rose corresponding to the PLD of larvae during March and April 2018 from 3 wind stations (Valley, Rhyl and Crosby). The direction is based on where the wind blow to. The wind strength (m/s) is represented by colour scale. The frequency is represented by the inner circle

For surface larvae, we found that the wind reduces the spatial variability in dispersal from the ten discrete North Wale mussel beds—in effect, ‘funnelling’ the larvae to similar settlement zones. Indeed, during the spring 2018 simulation, no significant difference between sites was observed on net transport variation. Corte et al. [13] showed that extreme weather could affect metapopulation organization. During spring 2018 a westerly wind persisted in the study region at 10 m/s for 15% of the time (week 2 in Fig. 7). These conditions could explain the large net dispersal distances (60 km) observed between weeks 1 and 2 (Fig. 3C), and why most larvae were concentrated in the same area (western Irish Sea). We suggest, therefore, that strong wind events can focus the connectivity of a large area of source mussel beds towards a single sink location.

**Fig. 8:**
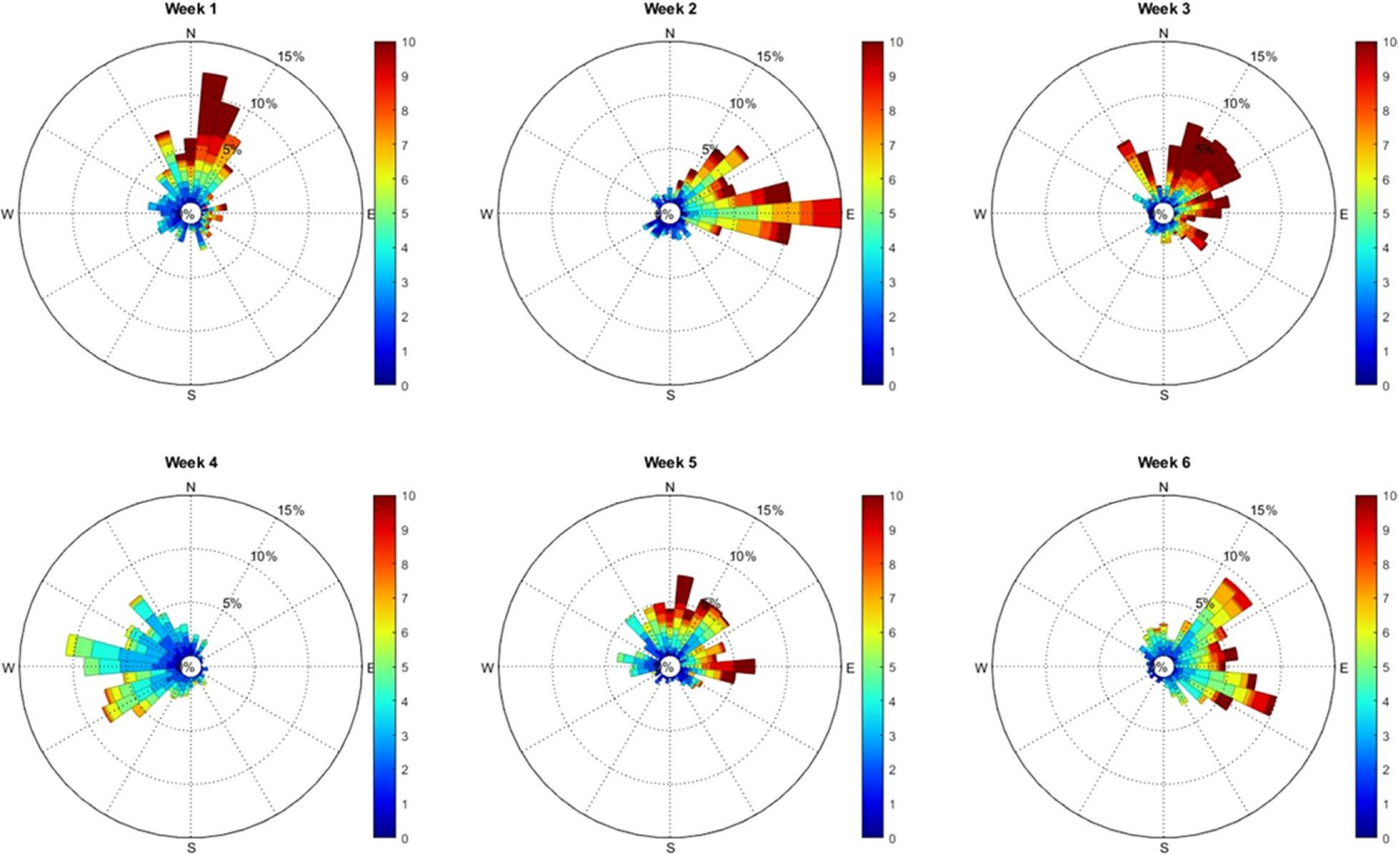
Wind rose corresponding to the PLD of larvae during March and April 2014 from 3 wind stations (Valley, Rhyl and Crosby). The direction is based on where the wind blow to. The wind strength (m/s) is represented by colour scale. The frequency is represented by the inner circle

In contrast, during the spring 2014 wind-driven simulation, larval dispersal was sensitive to both pelagic larval duration and source location. Interestingly, a persistent easterly wind occurred during this period that reversed the tidal residuals at the surface (Fig. 8). These differences in wind fields can explain the differences in simulated connectivity, while in spring 2014 the North Wales mussel beds were predominantly connected with the British coast (Morecambe Bay, the Cumbrian coast and Ribble estuary), and in spring 2018, the beds were predominantly connected with the Irish coast (Dundalk and Dublin). Furthermore, mussel farmers indicated there was good mussel recruitment in Morecambe Bay in 2014, which allowed them to harvest several tonnes of mussel seed, whereas in 2018 no seed where harvested in Morecambe Bay (Trevor Jones pers.comm.).

Our findings, coupled with mussel farmers observations suggest that the PTM represents to some extent mussel larval dispersal in the Irish Sea. It means that some *M. edulis* are potentially distributed in the upper water column and influenced by wind-driven currents [30, 50, 76]. Despite this study focuses on two contrasted year, it would be interesting to correlate more data from mussel farmers on harvest with wind forecast. In addition, our results suggest that the connectivity between commercial mussel bed located in North Wales and Morecambe Bay (i.e., the main source of spat for mussel shellfisheries) only exist under specific conditions: mussel travelling at the surface with a southwesterly wind such as that occurred in 2014. However, as we simulated five independent PLDs, from 2 to 6 weeks. Each with a competency period of one (final) week, our results for the longer PLD simulations could potentially over-estimate the connectivity, as larvae could have a longer competency period and hence settle before the final week. Weather variability between 2014 and 2018 might have influenced the timing of spawning and the amount of gamete released in the water column, which could have influenced observation on settlement made by mussel farmers in Morecambe Bay in 2018 and 2014 [4, 43].

Understanding interannual variability of mussel settlement and the possibility to predict where/when to collect mussel seeds are important for mussel aquaculture. However, settlement timing is important for connectivity and metapopulation resilience [70]. The results show that connectivity varied markedly through the 6-week pelagic phase. There are large uncertainties about mussel settlement patterns,no field experiments have been carried out to study where settled larvae come from and when they exactly settled. These uncertainties are due to a lack of studies on mussel larvae settlement. Indeed, it is challenging to study/observe settlement timing on relatively small organisms (< 1 mm). While our biophysical model helps circumvent this problem, other scientific methods—such as genetic and/or microchemistry—should supplement the modelling in the future to better understand mussel connectivity.

Whilst several studies have shown that increased PLD implies increased larval dispersal distance [25, 60, 67], we also showed that a change in the wind field can disrupt this. In our study, we show that dispersal distances are reduced after 3 weeks in 2018 because of a change in the wind patterns. Variable winds could therefore increase self-recruitment.

## Conclusion

Dispersal of mussel larvae in the tidally energetic Irish Sea during the April–May spawning season is potentially driven by wind-driven surface currents, based on model simulations of larval transport that include wind effects validated by shellfish farmer observations on recruitment in Morecambe Bay during summer 2014 and 2018. For the shellfish industries, there are two potential consequences: (1) spat should be collected near the surface to maximize the catch (e.g. from 0 to 5 m); and (2) it might be possible to predict the areas of larvae recruitment based on wind hindcasts or forecasts. Further, we estimate that North Wales mussel populations are well connected via larval transport, although with interannual variability that is contingent on wind variability. These results are important given the context of the Irish Sea, which is a hot spot for offshore renewable energy. Indeed, these offshore structures could act as artificial reefs with both beneficial (e.g. increase local biodiversity) and detrimental (e.g. habitat loss, spread of invasive species) impacts—hence spatial planning is crucial.

## Supplementary Information

**Additional file 1: Appendix 1**. **Table 1**: Model validation for: surface elevation showing RMSE (in m), NRMSE (in %) and coefficient of determination (R^2^); tidal analysis for M2 tidal constituent. **Table 2**: Model validation of simulated velocity direction and magnitude showing RMSE (in degree and m/s for direction and magnitude respectively), NRMSE (in %) and coefficient of determination (R^2^).

## Data Availability

The datasets used and/or analysed during the current study are available from the corresponding author on reasonable request.
