# Supplementary material for: The role of wind in controlling the connectivity of blue mussels (Mytilus edulis L.) populations
Source: Mov Ecol. 2022 Jan 21;10:3. doi: 10.1186/s40462-022-00301-0 (PMC8783501; doi:10.1186/s40462-022-00301-0)
Supplement: Supplementary file 1 — Additional file 1: Appendix 1. Table 1: Model validation for: surface elevation showing RMSE (in m), NRMSE (in %) and coefficient of determination (R2); tidal analysis for M2 tidal constituent. Table 2: Model validation of simulated velocity direction and magnitude showing RMSE (in degree and m/s for direction and magnitude respectively), NRMSE (in %) and coefficient of determination (R2). [file 40462_2022_301_MOESM1_ESM.docx]

**Appendix 1:**

Surface elevations and depth-averaged velocities computed from the 2018 simulation were compared against observations from 14 tide gauges ([www.ntslf.org](http://www.ntslf.org)) and seven offshore velocity moorings (data from Bangor University, Lewis *et al.*, 2017) (Figure 1). Model accuracy and skill was described using the Root Mean Square Error (RMSE in m); the Normalized Root Mean Square Error (NRMSE in %); and linear regression score (R^2^). Tidal analyses were performed on the model data, using the T_TIDE Matlab toolbox, and the principal semi-diurnal lunar tidal constituent (M2 amplitude) compared to observations at 16 sites (taken from Admiralty tidal stream atlas) with an average error of 4.3% (Table 1). Simulated tidal elevation had a mean error of 5.7% for the 14 sites (Table 1), and tidal current velocity had a mean error of 9.8% for magnitude and 11.2% for direction (Table 2).

Table 1: Model validation for: surface elevation showing RMSE (in m), NRMSE (in %) and coefficient of determination (R^2^); tidal analysis for M2 tidal constituent. The mean values among the sites are presented in bold.

| **Sites** | | **Surface elevation** | | | **Tidal analysis of M_2_** | | |
| --- | --- | --- | --- | --- | --- | --- | --- |
| **Id** | **Names** | **RMSE**  **(in m)** | **NRMSE (in %)** | **R^2^** | **Tide table data (m)** | **Model data (m)** | **Difference (%)** |
| 1 | Millport | 0.37 | 8.69 | 0.84 | 1.13 | 1.19 | 5.20 |
| 2 | Portpatrick | 0.33 | 6.97 | 0.91 | 1.34 | 1.35 | 0.90 |
| 3 | Bangor (N. Ireland) | 0.29 | 7.45 | 0.9 | - | - | - |
| 4 | Workington | 0.4 | 4.37 | 0.96 | 2.73 | 2.64 | -3.20 |
| 5 | Port Erin | 0.3 | 4.85 | 0.96 | 1.76 | 1.79 | 2.00 |
| 6 | Heysham | 0.72 | 6.93 | 0.92 | 3.17 | 2.82 | -11.10 |
| 7 | Liverpool | 0.69 | 6.88 | 0.92 | 3.12 | 2.71 | -13.10 |
| 8 | Llandudno | 0.42 | 4.77 | 0.97 | 2.67 | 2.36 | -11.60 |
| 9 | Holyhead | 0.28 | 4.48 | 0.96 | 1.81 | 1.73 | -4.40 |
| 10 | Barmouth | 0.35 | 6.95 | 0.9 | 1.47 | 1.44 | -2.30 |
| 11 | Milford haven | 0.37 | 4.92 | 0.96 | 2.24 | 2.13 | -4.70 |
| 12 | Hinklepoint | 0.35 | 2.86 | 0.99 | 3.8 | 3.83 | 0.90 |
| 13 | Ilfracombe | 0.23 | 2.31 | 0.99 | 3.08 | 3.01 | -2.40 |
| 14 | Amlwch | - | - | - | 2.3 | 2.25 | -2.20 |
| 15 | Beaumaris | - | - | - | 2.54 | 2.37 | -6.70 |
| 16 | Menai Bridge | - | - | - | 2.33 | 2.26 | -3.10 |
| 17 | Caernarfon | - | - | - | 1.61 | 1.41 | -12.50 |
| 18 | Portbury | 1.16 | 8.04 | 0.74 | - | - | - |
| **Mean** | | **0.45** | **5.75** | **0.92** |  |  | **- 4.27** |

Table 2: Model validation of simulated velocity direction and magnitude showing RMSE (in degree and m/s for direction and magnitude respectively), NRMSE (in %) and coefficient of determination (R^2^). The average values among the sites are presented in bold.

| **Sites** | | **Velocity direction** | | | **Velocity magnitude** | | |
| --- | --- | --- | --- | --- | --- | --- | --- |
| **Id** | **Names** | **RMSE (degree)** | **NRMSE (%)** | **R^2^** | **RMSE (m/s)** | **NRMSE (%)** | **R^2^** |
| 19 | North Hoyle | 41.44 | 11.72 | 0.82 | 0.04 | 5.75 | 0.97 |
| 20 | Conwy Bay | 37.35 | 10.53 | 0.86 | 0.04 | 9.01 | 0.96 |
| 21 | Red Wharf Bay | 46.12 | 13.1 | 0.75 | 0.06 | 12.42 | 0.84 |
| 22 | Brick works | 18.09 | 10.14 | 0.95 | 0.11 | 7.38 | 0.92 |
| 23 | Rhydwyn | 17.15 | 6.34 | 0.95 | 0.1 | 9.44 | 0.88 |
| 24 | Newborough | 49.39 | 13.73 | 0.82 | 0.06 | 15.94 | 0.68 |
| 25 | Porth Colmon | 44.37 | 12.71 | 0.75 | 0.09 | 8.47 | 0.93 |
| **Mean** | | **36.27** | **11.18** | **0.84** | **0.07** | **9.77** | **0.88** |
